# Tyrosine kinase inhibitors and mesenchymal stromal cells: effects on self-renewal, commitment and functions

**DOI:** 10.18632/oncotarget.12649

**Published:** 2016-10-13

**Authors:** Adriana Borriello, Ilaria Caldarelli, Debora Bencivenga, Emanuela Stampone, Silverio Perrotta, Adriana Oliva, Fulvio Della Ragione

**Affiliations:** ^1^ Department of Biochemistry, Biophysics and General Pathology, Second University of Naples, Naples, Italy; ^2^ Department of Woman, Child and of General and Specialized Surgery, Second University of Naples, Naples, Italy

**Keywords:** tyrosine kinase inhibitors, target therapy, mesenchymal stromal cells, bone marrow milieu, osteogenesis

## Abstract

The hope of selectively targeting cancer cells by therapy and eradicating definitively malignancies is based on the identification of pathways or metabolisms that clearly distinguish “normal” from “transformed” phenotypes. Some tyrosine kinase activities, specifically unregulated and potently activated in malignant cells, might represent important targets of therapy. Consequently, tyrosine kinase inhibitors (TKIs) might be thought as the “vanguard” of molecularly targeted therapy for human neoplasias. Imatinib and the successive generations of inhibitors of Bcr-Abl1 kinase, represent the major successful examples of TKI use in cancer treatment. Other tyrosine kinases have been selected as targets of therapy, but the efficacy of their inhibition, although evident, is less definite. Two major negative effects exist in this therapeutic strategy and are linked to the specificity of the drugs and to the role of the targeted kinase in non-malignant cells. In this review, we will discuss the data available on the TKIs effects on the metabolism and functions of mesenchymal stromal cells (MSCs). MSCs are widely distributed in human tissues and play key physiological roles; nevertheless, they might be responsible for important pathologies. At present, bone marrow (BM) MSCs have been studied in greater detail, for both embryological origins and functions. The available data are evocative of an unexpected degree of complexity and heterogeneity of BM-MSCs. It is conceivable that this grade of intricacy occurs also in MSCs of other organs. Therefore, in perspective, the negative effects of TKIs on MSCs might represent a critical problem in long-term cancer therapies based on such inhibitors.

## INTRODUCTION

The major purpose of chemotherapy is to transform fatal cancers into manageable diseases with a long life expectancy. To this goal, it appears essential to selectively identify and target the lesions that induce and maintain cancer development, survival and metastasization. Malignancies result from the acquisition of somatic, both genetic and epigenetic, alterations [1]. Myriad basic and preclinical studies have allowed the identification of functional changes that initiate and maintain a transformed phenotype. Although the number of these alterations has been usually estimated as more than 30, making difficult to reach cancer eradication, in few cases it has been possible to identify the genetic/epigenetic changes having “driver” relevance [2].

These key alterations frequently involve tyrosine kinase (TK) activities, both associated to growth factor receptors or to non-receptor intracellular proteins [3]. Thus, strong efforts in cancer treatment have been directed to synthesize molecules with specific capability of inhibiting altered TKs, responsible for or associated to cancer transformation [3, 4]. However, two main negative aspects appear linked to TK targeted therapy. One is related to the frequent intrinsic absence of specificity of these compounds, generally designed to compete with ATP for binding to the enzyme active site. The second is the role that the targeted tyrosine kinases play in non-malignant tissues, which might be the cause, during the treatment, of remarkable negative side effects.

Although the identification and prevention of undesired negative outcomes due to TK inhibitor (TKI) chemotherapy represent main topics of the clinical research, few studies have focused on the analysis of the TKIs effects on MSCs, one of the most heterogeneous and widely distributed cell populations in human body. This review summarizes the key roles of MSCs in human physiology and discusses the data available on the TKI activities on MSCs. We suggest that the complex effects of TKIs on cell functions (embracing cell growth, differentiation and death) might strongly affect MSC outcomes, as control of bone turn-over and hemopoiesis. Therefore, long-term safety data on important chemotherapy agents, as TKIs are, should necessarily include a clear knowledge of their effects on human MSCs.

## TYROSINE KINASE INHIBITORS

Targeted cancer therapy (TCT) agents refer to a heterogeneous group of molecules able to inhibit the growth and progression of malignant cells by interfering, differently from conventional cytotoxic chemotherapeutics, with specific signalling pathways usually up-regulated in cancer cells and supposed to be involved in the malignant status [3, 5, 6].

One of the most promising categories of TCT molecules includes compounds targeting kinases. Protein phosphorylation is the most common reversible post-translational modification [7], with an estimated 50% of all proteins undergoing phosphorylation. There are more than 500 kinases including about 90 TKs of both receptor- and non-receptor type, encoded by the human genome, and, despite the very ample number of studies in the field, the underlying function of many of these enzymes remains to be elucidated [8]. Today, nearly 30 drugs inhibiting different protein kinases have been approved for cancer treatment and several additional therapeutics are at different phases of clinical evaluation. Some of these compounds are reported in Table 1.

**Table 1:** MAJOR TK INHIBITORS TARGETING CANCER-ASSOCIATED TKs

Name	Main Target(s)	FDA Approval	Indication(s)
Bcr-Abl1 Inhibitors			
Imatinib	BCR-Abl1, Abl, KIT, PDGFRs	2001	CML, GIST
Dasatinib	BCR-Abl1, Abl, Src, Kit, PDGFRs, EPH, CSK	2006	CML, ALL
Nilotinib	BCR-Abl1, Abl, Kit, Lck, EPHA3, DDR1	2007	CML
Bosutinib	BCR-Abl1, Abl, Src, Lyn, Hck	2012	CML
Ponatinib	BCR-Abl1, Abl, PDGFRα, Src, KIT, FGFR, VEGFRs	2012	CML, Ph+ ALL
EGFR Inhibitors			
Erlotinib	EGFR	2004	NSCLC, pancreatic cancer
Gefitinib	EGFR	2005	NSCLC, AML
Afatinib	HER2, EGFR, T790M mutated EGFR	2013	NSCLC, squamous cell carcinoma of the head and neck, breast cancer
Osimertinib	EGFR, T790M mutated EGFR	2015	NSCLC
Dacomitinib	EGFR	--	NSCLC, gastric cancer, head and neck cancer, glioma
Rociletinib	EGFR, T790M mutated EGFR	--	NSCLC
HM61713	EGFR, T790M mutated EGFR	--	NSCLC
ASP8273	EGFR, T790M mutated EGFR	--	NSCLC
EGF816	L858R, Ex19del, and T790M mutated EGFR	--	NSCLC
PF-06747775	EGFR, T790M mutated EGFR	--	NSCLC
Alk Inhibitors			
Crizotinib	ALK, MET	2011	ALCL, NSCLC, Neuroblastoma
Ceritinib	ALK	2014	NSCLC
Alectinib	ALK	2015	NSCLC
Brigatinib	ALK, EGFR	--	ALCL, NSCLC, Neuroblastoma
CEP-28122/CEP-37440	ALK	--	ALCL, NSCLC
Entrectinib	TrkA, TrkB, TrkC, ROS1, ALK	--	Neuroblastoma
PF-06463922	ROS1, ALK	--	NSCLC
TSR-011	ALK, TRK	--	NSCLC
X-376/X-396	ALK	--	NSCLC
HER/ErbB Receptor Inhibitors			
Lapatinib	HER2	2007	Breast Cancer
Neratinib	HER2	--	Breast Cancer
VEGFR and PDGFR Inhibitors			
Sorafenib	VEGFR, PDGFR and Raf kinases	2005	Renal cell carcinoma, hepatocellular carcinoma, iodine resistant advanced thyroid carcinoma
Sunitinib	PDGF-Rs, VEGFRs	2006	Kidney cancer, GIST, pancreatic neuroendocrine tumors
Pazopanib	c-KIT, FGFR, PDGFR and VEGFR	2009	Renal cell carcinoma, soft tissue sarcomas
Vandetanib	VEGFR. EGFR, c-Ret	2011	Unresectable, locally advanced, or metastatic medullary thyroid cancer
Sunutinib	VEGFR, KIT, PDGFR	2011	Pancreatic neuroendocrine tumors, kidney cancer, GIST
Pazopanib	VEGFR, KIT, FGFR, PDGFR	2012	Renal cell carcinoma, soft tissue sarcomas
Regorafenib	VEGFR1, VEGFR2, VEGFR3, KIT, PDGFR FGFR1, FGFR2,	2012	Metastatic colorectal cancer, GIST
Cabozantinib	VEGFR2, c-Met	2012	Thyroid cancer, advanced renal cell carcinoma, prostate cancer, glioblastoma multiforme
Axitinib	VEGFR1, VEGFR2, VEGFR3	2012	Renal cell carcinoma, CML
Lenvatinib	VEGFR1, VEGFR2, VEGFR3, PDGFRs, FGFRs, c-Kit, RET,	2015	Thyroid cancer
Linifanib	VEGFR, PDGFR	--	NSCLC, liver cancer, breast cancer, colorectal cancer
Bruton's tyrosine kinase Inhibitor			
Ibrutinib	Bruton's tyrosine kinase	2013	CLL, mantle cell lymphoma, B-cell malignancies

CML, Chronic Myelogenous Leukemia; GIST, Gastrointestinal Stromal Tumour; ALL, Acute Lymphocytic Leukemia; Ph+ ALL, Philadelphia positive ALL; NSCLC, Non Small Cell Lung Carcinoma; AML, Acute Myelogenous Leukemia; ALCL, Anaplastic Large Cell Lymphoma

The quintessence of TKIs is, beyond any doubts, represented by imatinib mesylate (IM, or STI-571, marketed as Gleevec or Glivec). IM was synthesized as a small molecule able to bind to the ATP-binding site of tyrosine kinases, and then, identified as a highly specific and powerful inhibitor of BCR-Abl1 kinase activity. Therefore, it was introduced in the treatment of chronic myelogenous leukemia (CML). CML origins from a 9:22 chromosome balanced translocation, which gives rise to the so-called chromosome Philadelphia and to the constitutively activated fusion BCR-Abl1 tyrosine kinase. IM was approved by FDA on May 10, 2001 [9] and its introduction in therapy has enormously ameliorated the prognosis of CML, prolonging patient's survival and decreasing disease recurrence.

As a matter of fact, IM changed the scenery of CML treatment, improving the CCyR (complete cytogenetic response) from 10%-25% to 80%-95%, and the 10-year overall survival from 10%-20% to 80%-90% [10–12]. IM has also been shown efficacious in the inhibition of c-Kit, and therefore it has been introduced in therapy of gastrointestinal stromal tumour [13, 14]. Other tyrosine kinases inhibited by IM are c-fms, PDGFRα and PDGFRβ [15].

However, although IM remains the gold standard of CML first-line treatment, in many cases resistance or intolerance to the drug emerge. In particular, the resistance might be ascribed to four major events: i) gene amplification or mutations at the kinase site; ii) decreased intracellular IM levels due to enhanced activity of drug exporters and activation of alternative pathways functionally compensating for IM-sensitive mechanisms; iii) induction of immature CML cell quiescence, and iv) suppression of BCR-Abl1 expression [16–21].

**Figure 1:**
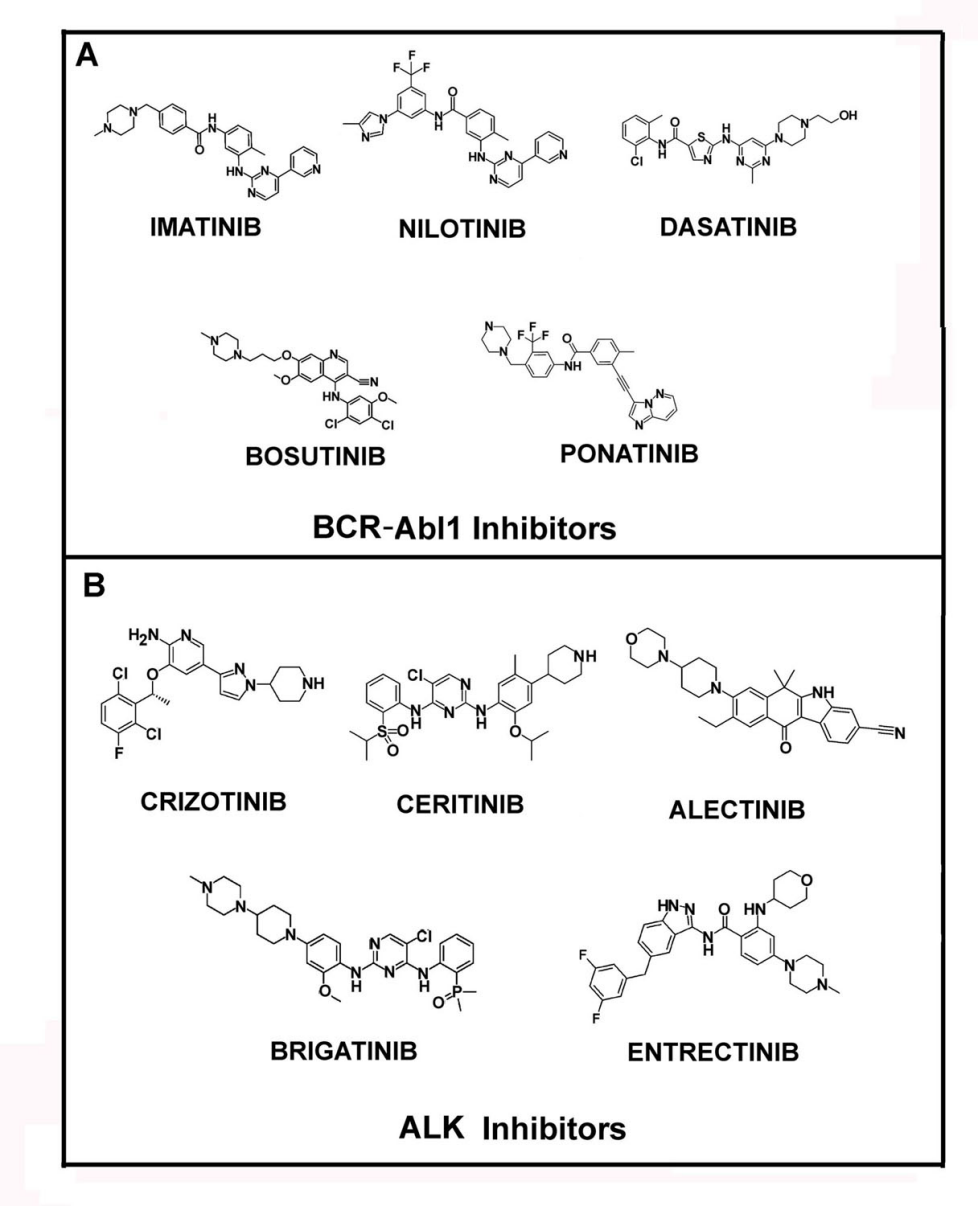
Structures of Bcr-Abl1 and ALK inhibitors In panel A the molecular structures of main Bcr-Abl1 inhibitors, Imatinib, Nilotinib, Dasatinib, Bosutinib and Ponatinib are reported. In panel B the structures of ALK inhibitors, Crizotinib, Ceritinib, Alectinib, Brigatinib and Entrectinib, are shown.

Moreover, advanced phase CML shows remarkably reduced response rates to IM monotherapy, with relapse being common within a year. This has lead to the development of second (nilotinib, dasatinib and bosutinib) and, more recently, third generation (ponatinib) TKIs (Figure 1A) [22].

**Figure 2:**
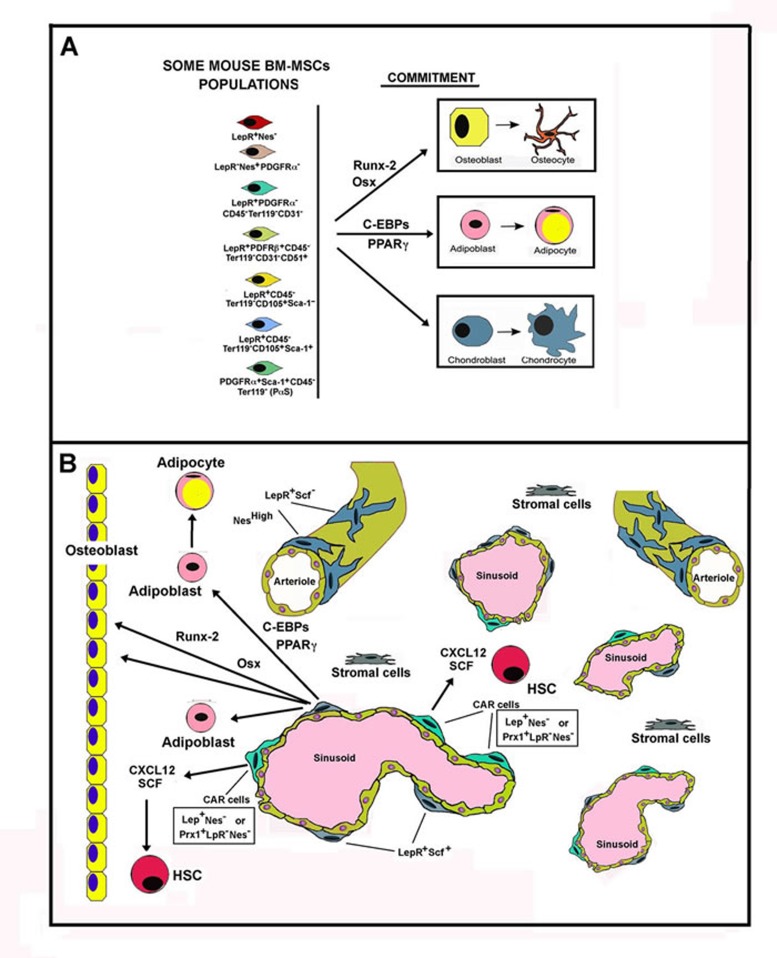
Schematic representation of Mouse Bone Marrow MSC populations Panel A. The figure shows, on the left, some mouse BM-MSC populations with regards to Leptin Receptor (LepR) occurrence. On the right, the figure reports the master genes activated during the commitment of MSCs towards the osteogenic, adipogenic and chondrogenic differentiation. Panel B. The figure reports some cell populations occurring in bone. On the right are reported osteocytes and osteoblasts that concur to the formation of osteid matrix. The other part of the panel depicts some cell populations present in bone marrow. HSC means hemopoietic staminal cells. Observe the localization of CAR cells near the sinusoids and their strict relationship to HSCs. CAR cells are the major source of CXCL12 and SCF, factors that are required for HSCs differantiation occurring in the hemopoietic niche. Osteoblasts also produce soluble factors required for HSCs commitment (showed as an arrow).

Additional TKIs have been developed and introduced in therapy. Similarly to the TKIs directed towards BCR-Abl1, at least three generations of anti-EGFR (Epidermal Growth Factor Receptor) tyrosine kinase inhibitors exist. The first-generation include gefitinib and erlotinib, which are effective in the first line treatment of advanced non-small cell lung cancer (NSCLC) harboring activated EGFR mutations [23–25]. The second-generation of EGFR-targeting TKIs, afatinib and dacomitinib, irreversibly bind to the tyrosine kinase domain of EGFR and of other ErbB-family members [26, 27]. EGFR targeting is now the standard therapy for NSCLC patients with EGFR activating mutations, but nearly 50-60% of the treated cases develop resistance to the drug by introducing a T790M mutation. This genetic change has required the development of the third generation of EGFR-directed TKIs, that includes AZD9291 (osimertinib or mereletinib), CO-1686 (rociletinib), HM61713, ASP8273, EGF816, PF-06747775, all being effective in the therapy of NSCLC harboring mutated EGFR [28]. The HER (ErbB) TK receptors are also implicated in other cancers for which additional anti-HER therapeutics have been approved, including lapatinib and neratinib [29–31].

The treatment of patients harboring chromosomal rearrangements of ALK (anaplastic lymphoma kinase) was revolutionized by crizotinib (approved by FDA in 2011), a small molecule inhibitor of ALK, ROS1 (c-ros oncogene 1) and c-MET (Figure 1B). Unfortunately, generally within the first 12 months, the ALK-dependent diseases progressed, due to further ALK mutations, and the patients develop crizotinib resistance [32–35]. Thus, in the last few years novel potent ALK inhibitors have become available, including: ceritinib (LDK378), brigatinib (AP26113), alectinib (RG7853/AF-802/RO5424802/CH5424802), entrectinib (RXDX-101, NMS-E628), PF-06463922, ASP3026, TSR-011, X-376/X-396 and CEP-28122/CEP-37440 (Figure 1B) [36].

Table 1 reports a list of the TKIs introduced in cancer therapy so far and some compounds that could be approved by FDA in the next years. It is evident that none of them is completely specific. This is an intrinsic problem of these drugs that appears difficult to overcome.

Specific classes of TKIs target specific receptors and they are typically associated with particular toxicities. For example, the use of EGFR inhibitors is associated with frequent skin rash, diarrhea, mucositis [37, 38]. In a smaller number of cases, pneumonitis has been observed. Different types of skin rash can occur. They most include acneiform or pustular rashes, although rashes can also show a more generalised distribution and maculopapular features [37, 38]. In addition, rash can be induced by sun exposure, thus appearing as photosensitivity rash. The most frequent rash associated with EGFR inhibition, the aracneiform type, is improved when medication is continued. Moreover, it resolves when treatment is interrupted [37–39]. The inhibition of VEGFR results in proteinuria, hypertension, some wound healing complications, and hand-foot skin reaction. Moreover, the use of TKIs directed against VEGFR causes some vascular complications, including left ventricular dysfunction and arterial thromboembolism. In particular, hypertension is the most frequent cardiovascular negative effect associated to treatment with several VEGFR inhibitors, namely axitinib and lenvatinib. Generally, it was observed quite early, within three to four weeks from starting of treatment [37–39].

Gastrointestinal toxicities are most commonly associated to the inhibition of ALK. The signs include nausea, vomiting and diarrhea [40–42]. In addition, alterations of some laboratory parameters are observed, including the increase of aspartate aminotransferase (AST) and alanine transaminase (ALT) [40–42]. It is to underline that increased AST and ALT is a common side effect during the treatment with TKIs. Accordingly, monitoring liver enzymes is clinically indicated throughout treatment.

Dose adjustments are helpful and can generally ameliorate the observed side effects. However, some complications occur very frequently, like pneumonia and pneumonitis during ceritinib treatments. However, whether such negative effects are linked to the treatment or are symptoms of the disease (lung cancer) appear in some cases difficult to establish [40–42].

Finally, also BCR-Abl1 inhibition typically causes several negative side effects, including cytopenia, hypothyroidism and cardiac abnormalities [43–46]. Furthermore, IM has been reported to induce, in patients subjected to a long-term therapy, a decrease of hematic phosphate levels (hypophosphatemia) associated to phosphaturia, and alterations of the bone turn-over markers. In particular, a decrease of serum osteocalcin and urine N-telopeptide was demonstrated [47–49]. Hypophosphatemia has also been reported as a side effect of nilotinib treatment, suggesting a plausible effect on the cell populations involved in bone metabolism, namely osteoblasts and osteoclasts [50].

To date, whether other TKIs affect bone turn-over is not described, yet; however, as reported in the next paragraphs, it is hypothesizable that long-term treatment migh have an impact on the major source of bone-synthesizing cells, namely BM-MSCs.

## MESENCHYMAL STROMAL CELLS

In 1987 Friedenstein *et al* first described MSCs, when they isolated from rat BM plastic-adherent, non-hematopoietic, fibroblast-like colony-forming cells (named colony-forming unit-fibroblast, CFU-F) which could be induced to differentiate into osteogenic, adipogenic and chondrogenic cells [51]. Subsequently, Caplan, after an intense debate, named them mesenchymal stem cells, abbreviated in MSCs, a definition that was widely diffused among the scientific community [52]. In 2005, due to the absence of highly specific markers and the heterogeneity of the cell population, the Mesenchymal and Tissue Stem Cell Committee of the International Society for Cellular Therapy proposed to use the definition of “multipotent mesenchymal stromal cells”, maintaining the acronym MSC regardless of the tissue from which they were isolated [53]. Conversely, the term of mesenchymal stem cells should be reserved to a subpopulation of these cells, endowed with “stemness” demonstrated by defined criteria. In 2006, the same Commission clearly stated the following minimal criteria to which MSCs must obey: (i) adherence to plastic under standard culture condition; (ii) expression of cell surface markers such as CD73, CD90, and CD105 and lack of expression of CD34, CD45, CD14 or CD11b, CD79 or CD19, and HLA-DR, and (iii) capacity to differentiate into osteoblasts, adipocytes, and chondroblasts [54]. In the last years, as we will discuss in details later, several additional markers have been associated with MSCs. In this review we will refer to multipotent mesenchymal stromal cells as MSCs, unless otherwise stated.

Today, MSCs can be easily obtained from many tissues other than BM, such as umbilical cord, placenta, adipose tissue (AT), skeletal muscle, tendon, trabecular bone, dentary pulp and skin, lung and liver. BM and AT, however, represent the major MSC sources [55, 56], although fundamental differences, in terms of phenotypic properties, have been identified between AT-MSCs and BM-MSCs [57].

The relevance of MSC use in the area of tissue transplantation and regenerative medicine is growing exponentially, due to some important characteristics, which include: i) self-renewal capacity; ii) ability to migrate to the site of injury; iii) potentiality to differentiate towards the major mesenchymal tissue phenotypes, mainly bone and cartilage, and iv) immunomodulatory and anti-inflammatory properties. MSCs are also able to release cytokines and factors influencing cell survival and proliferation, such as heparin epidermal growth factor (HB-EGF), basic fibroblast growth factor (bFGF), platelet-derived growth factor-B chain (PDGF-B), vascular endothelial growth factor (VEGF), keratinocyte growth factor (KGF), and angiopoietins [58], which are all well known enhancers of tissue repair *in vivo* [58].

In addition to the increasing interest in developing methods for MSC isolation and *in vitro* expansion and in their use in biomedical applications, numerous studies focused on MSC physiological role in specific tissues. In BM, MSCs constitute only a little fraction of nucleated cells, representing one of the key components of BM microenvironment and playing at least three major roles in BM physiology. One is linked to their differentiation into osteoblasts and, in turn, to the general control of bone turnover and fracture healing. Also, physiological MSC differentiation towards adipocytes is emerging, underlying the function of BM fat in marrow function. The second major role of hBM-MSCs is the participation into the hematopoietic niche organization and thus in the blood cell origin process. In the niche microenvironment, BM-MSCs play a key role in hematopoietic stem cell commitment, mobilization, and exit from BM. Finally, BM-MSCs exert important immunomodulatory functions participating to the control of both adaptive and innate immune response.

### BM-MSC populations and lineage tracing studies

In the last few years, the variety of MSC roles in several physiological/pathological processes has prompted an enormous interest on this cell population. Consequently, several pivotal studies on MSC characterization have been published. On the other hand, numerous controversies on MSCs embryonic origin exist and their phenotypic characterization still needs to be fully unraveled. The use of genetic drivers in transgenic animals (i.e. mice) has enormously ameliorated MSC characterization and thorough investigations have gradually contributed to shed light on such a complex issue. Among the various MSC populations, those localized in BM have been the most exhaustively investigated, particularly for their participation to the hematopoietic niche. Therefore, the following description is mainly related to BM-MSCs. It is however to be taken into account that MSCs occur in all tissues, generally in perivascular position, but their characterization is so far less accurate. These extra-BM MSC populations have been correlated to degenerative processes (such as fibrosis) and their targeting might be important in the development of TKIs side effects.

BM-MSC immunophenotypes are strongly heterogeneous, suggesting that these cells include several distinct sub-populations, all sharing the *in vitro* ability to differentiate, under enforced conditions, toward different specific phenotypes (osteoblasts, adipocytes and chondroblasts). At the present, the availability of persuasive approaches for lineage tracing, along with reliable immunophenotypical characterization, have allowed conclusions on an extensive BM-MSCs heterogeneity. Two key methods of cell lineage tracing have been developed in recent years [59–62]. The first is based on the use of chimeras in which a genetic marker (like green fluorescent protein, GFP, yellow fluorescent protein, YFP, or other naturally fluorescent proteins) is expressed under the control of a cell-type specific promoter. This approach allows the identification and characterization of cell populations expressing the gene controlled by the selected promoter [59–62]. The other experimental approach is more complex and permits to follow cells transcribing a selected gene in a specific time window during embryogenesis or post-natal life; it uses the Cre recombinase, whose expression is conditionally activated to stably label a cell type and its descendants [59–62]. More precisely, Cre recombinase is placed under the control of a particular promoter and thus is expressed in a specific phenotype. Then, Cre recombinase expression allows the transcription of a marker that traces cells and their derivatives. The technique has been further ameliorated by additional genetic handlings that allow the expression of Cre recombinase only after the administration to the transgenic mice of a specific molecule (for example, tamoxifen) [61, 62]. Therefore, in such Cre-inducible mice, it is possible to activate the tracing of a specific cell lineage in a selected time-window (either in embryonic or post-natal life). By means of these approaches important advancements in BM-MSCs studies have already been obtained. Further lineage tracing studies are nevertheless required to obtain a definite picture of the mouse BM-MSC sub-populations.

Several markers have been so far identified that allow a putative identification of mesenchymal stromal cells. BM-MSCs do not express the following markers: CD34, CD45 (Protein tyrosine phosphatase, receptor type C), CD31 (Platelet endothelial cell adhesion molecule, PECAM-1), and the co-stimulatory proteins CD40, CD80 and CD86 [63]. All these CDs are characteristic of hemopoietic staminal cell (HSC) phenotypes. Conversely, CD105 (endoglin, a TGF-βR accessory molecule also called TGF-βRIII) and CD146 (melanoma cell adhesion molecule, MCAM, or cell surface glycoprotein MUC18, that is a receptor for laminin alpha 4) are expressed on BM-MSCs. Other reported MSC CD markers include: CD73 (ecto-5-endonucleotidase), CD44 (homing cell adhesion molecule), CD90 (receptor for the Fc region of IgA, THY-1), CD71 (transferrin receptor), CD166 (activated leukocyte cell adhesion molecule), CD29 (integrin beta-1), CD9 (integrin interacting protein), CD13 (aminopeptidase N), CD106 (VCAM-1), CD54 (ICAM-1), CD3 (T-cell co-receptor), integrins (CD49, CD11 and β4 integrins), GD2 Ganglioside, CD271 (low affinity nerve growth factor receptor) and others [63, 64]. However, the CD45^−^ CD31^−^CD105^+^CD146^+^ cell population has been reported to include all mouse BM-MSCs with CFU-F (Colony-Forming-Fibroblast-Units) ability and capable of generating mesenspheres (i.e. cells growing as nonadherent spheres), two major inherent MSCs features [63, 64].

In addition to the markers listed above, others need to be considered with peculiar attention, namely: nestin (Nes), receptor of leptin (LepR), stem cell factor (SCF), C-X-C motif chemokine ligand 12 (CXCL12), neural/glial antigen 2 (NG2), paired related homeobox 1 protein (Prx1), myxovirus resistance-1 (Mx1), platelet-derived growth factor receptor α (PDGFRα or CD140a), osterix (Osx), Runt-related transcription factor 2 (Runx2) and collagen type II (Col2) [65–75].

BM-MSC subpopulations expressing different patterns of these proteins have been putatively identified, making tremendously complicated a straightforward MSC characterization. SCF and CXCL12 are soluble factors produced by BM mesenchymal sub-population required for HSC maintenance in the hematopoietic niche, while Prx1 is a DNA-interacting protein whose expression occurs only in the mesoderm during embryonic development and only in mesenchymal tissues in adult mice [68, 70].

An additional consideration should be made before discussing further the heterogeneity of BM-MSCs. In relation to the different calcification processes, MSCs can have two distinct germ layer derivations, from mesoderm or from neural crest. Particularly, craniofacial bones are generated by the neuroectoderm (neural crest) through intramembranous ossification, whereas the axial and appendicular bones are derived from paraxial and lateral mesoderm through endochondral ossification.

We will discuss some of the data available in Literature considering the most intriguing markers, but also having in mind that no conclusive data have been reached.

Nes is an intermediate filament protein (also named Type VI intermediate filament protein). The protein is particularly expressed in neuronal cells where it is involved in the axon growth. GFP expression driven by Nes promoter in transgenic mice (Nes-GFP mice) strongly mark hair follicle stem cells that, *in vitro*, might differentiate into various phenotypes, including glia, neurons, smooth muscle cells, keratinocytes and melanocytes.

In 2013, a study on Nes positive (Nes^+^) and negative (Nes^−^) BM-MSCs, based on transgenic Nes-GFP mice, was published [76]. The auhors identified two Nes^+^ BM-MSC populations, evidencing a clear distinction between Nes^+^ cells localized near arterioles and near sinusoids. Arterioles are generally surrounded by layers of smooth muscle cells and receive innervation by sympathetic nerves. An MSC population, expressing high Nes level and occurring along arterioles [76] was found, from a quantitative point of view, quite scarce (about 0.002% of all BM cells) [76]. Alternatively, a different BM-MSC population expresses lower level of Nes, is more abundant, reticular in shape and generally associated with sinusoids. One year later, two studies analyzed the origin, role and fate of Nes^+^ cells during BM niche formation and endochondral calcification [77, 78]. In these studies, two tracing approaches were employed based on the use of, alternatively, Nes-GFP mice or Nes-Cre^ER^ mice (ER stands for estrogen receptor). The latter model, in particular, traces cells targeted by tamoxifen-inducible Cre recombinase driven by Nes enhancer. In Nes-GFP mice, Ono and colleagues evidenced different endothelial and non-endothelial Nes^+^ cells in the embryonic long bone perichondrium [77]. Specifically, the non-endothelial Nes^+^ cells acted as early precursors of osteoblasts and their differentiation was associated to the activation of IHH (Indian hedgehog) production and the expression of Runx2. These Nes^+^ osteoblasts (probably deriving from chondroblasts) constitute the initial ossification centers early occurring during the endochondral calcification process. Thus, a major contribution of this study was the identification of Nes^+^ osteoblast precursors (confirmed by the contemporaneous expression in some of these cells of osterix and Runx2) [77]. In postnatal bones, on the other hand, Nes^+^ cells are more heterogeneous and include a range of cells of the osteoblast and endothelial lineage. In addition, differences were evidenced between the two mouse models, in that, in developing BM, Nes-Cre^ER^ cells were essentially endothelial cells [77]. The authors also showed that Nes-GFP signal was remarkably intense in two sites: perivascular cells in the primary spongiosa, immediately near the growth plate, and in the pericytes of the arterioles.

An intriguing study on this issue, published in the same year (2014) reached partially different conclusions [78]. The authors observed (employing Nes-GFP mice) that GFP positive cells were present in BM of E16.5 embryos, mainly associated with blood vessels and they were mostly BM-MSCs and only scarcely endothelial cells. However, differently from the Ono's paper [77], these Nes^+^ BM-MSCs were not osteoblasts or chondroblasts [78]. In brief, the authors suggested that BM Nes^+^ cells do not contribute to fetal endochondrogenesis. A second conclusion of this study was that fetal endochondral skeletogenesis essentially involves mesoderm-derived Nes^−^ BM-MSCs; however, these cells loose their activity after birth. Conversely, initially quiescent and neuronal crest-derived Nes^+^ cells preserve their MSC activity (including the osteogenic capability), as well the possibility of participating to the hematopoietic niche (by producing CXCL12). Importantly, the authors also proposed that the expression of master regulators of chondrogenesis, osteogenesis, and adipogenesis progressively increase in postnatal Nes-GFP^+^ BM-MSCs. In summary, after birth, the niche-forming MSCs share an origin similar to peripheral neurons and glial cells, but completely distinct from BM-MSCs participating in the fetal endochondrogenesis and HSC niche formation [78]. Finally, a recent investigation has reported the occurrence of Nes^+^ BM-MSCs lacking the expression of both LepR and PDGFRα [66].

A second marker, that has been object of intensive investigation, is LepR. LepR is expressed on populations of neuron in the hypothalamus and most of leptin's known effects result from this central signaling. Previous studies concluded that it is the central activity of leptin that influences osteoblast functions and bone mass; however, due to the systemic nature of these studies, other confounding factors, such as global alterations in metabolism, could not be ruled out as contributing to bone phenotypes.

About 0.3% of BM-MSCs have been reported LepR^+^ and 10% of LepR^+^ cells are CFU-Fs, accounting for more than 90-95% of BM CFU-Fs. The level of LepR increases after birth in BM cells. Although mesenspheres have been reported to be LepR^+^ and Nes^+^, the occurrence of both markers on the same cell has been debated and LepR^+^ cells appeared more abundant than Nes^+^ cells [66]. Given their frequency, LepR^+^ cells have been indicated as the major origin of bone cells and adipocytes in adult BM, as well as a key source of HSC niche factors [66]. Indeed, it has been reported that the main component of the perivascular niche, i.e. CXCL12-abundant reticular (CAR) cells, are all LepR^+^ cells while being Nes^−^ [66]. Importantly, CARs are identified primarily around the sinusoids, where cells with a scarce (or a complete absence) of Nes have been found. In addition, it has been reported that LepR^+^ MSCs localized around sinusoids express SCF, while periarteriolar LepR^+^ MSCs are SCF^−^ [66].

In relation to the presence or absence of LepR, several different MSCs phenotypes in mice have been identified, including (among others): LepR^+^Nes^−^, LepR^−^Nes^+^PDGFRα^−^, LepR^+^PDGFRα^+^CD45^−^Ter119^−^CD31^−^, LepR^+^PDGFRβ^+^CD45^−^Ter119^−^CD31^−^CD51^+^, LepR^+^CD45^−^Ter119^−^CD105^+^Sca-1^+^, LepR^+^CD45, Ter119^−^ CD105^+^Sca-1^−^ and PDGFRα^+^Sca-1^+^CD45^−^Ter119^−^ (PαS) cells [66].

The analysis of NG2-Cre^ER^-expressing cells in mice suggest that NG2^+^ BM-MSCs are very scarce representing about 2% of CFU-F colonies formed by BM cells. A small amount of these NG2^+^ BM-MSCs express PDGFRα (phenotype NG2^+^PDGFRα^+^), while no NG2^+^PDGFR^+^SCF^+^ cells were reported [72]. Conversely, antibodies directed against NG2 stained chondrocytes and collagen-expressing osteoblasts and osteocytes [66]. Analogously, the occurrence of PDGFRα^+^ in CAR cells (CXCL12^+^ or SCF^+^ cells) has not been detected [66].

A recent study has characterized PDGFRα expression in human BM stromal cell population. The authors suggest that PDGFRα (CD140a) is scarcely expressed in adult human BM non-hematopoietic cells and conclude that CD140a is a key negative marker for adult human BM-MSCs, which might be useful for isolating population of adult mesenchymal stem/progenitor cells with remarkable hematopoiesis-supporting capacity [72]. This is in partial contrast with a different study reporting that human fetal BM-MSCs are PDGFRα^+^ [79]. If both the studies [72, 79] are correct, PDGFRα expression is regulated during development, being high in fetal BM-MSCs and scarce in adult BM-MSCs.

The analysis of cells expressing Cre recombinase driven by the Col2 promoter/enhancer has demonstrated that these cells contribute not only to chondrocytes, but also to early perichondral precursors, osteoblasts, CAR cells and BM stromal progenitor cells. In addition, it suggested that early Col2-Cre^ER^ marked postnatal cells (i.e. chondroblasts) furnished multiple mesenchymal lineages and their descendants for over a year [80]. Conversely, Cre/Cre^ER^ recombinases mice, driven by the Osx promoter, delineated a descendent population characterized by a limited lineage potential, in that it was able to generate only osteoblasts and stromal cells [80].

A further piece of the complexity in the BM-MSCs picture came from short-term ablation of CAR cells. This experimental strategy was obtained by using a gene ablation method defined as “toxin receptor-mediated cell knockout” [81] and, in particular by generating mice in which Diphtheria Toxin Receptor-GFP (DTR-GFP) fusion protein was inserted into the CXCL12 locus (CXCL12-DTR-GFP mice) [82]. In these mice the transcription of CXCL12 gene resulted in the expression of DTR and thus in ablation of cells expressing the gene after diphtheria toxin administration [82]. Conversely, wild-type murine cells were insensitive to diphtheria toxin killing. CAR cells removal did not affect the number of bone lining osteoblasts and endothelial cells. Intriguingly, the removal severely altered the differentiation potential (adipogenic and osteogenic commitment) of BM-MSCs, the synthesis of SCF and CXCL12 and strongly reduced lymphoid and erythroid progenitors. The study concluded that niche adipo-osteogenic progenitors are required for maintenance of undifferentiated hematopoietic stem cells and for growth of HSCs and lymphoid and erythroid progenitors [82].

In conclusion, the data reported and several additional studies not discussed here for space limit, clearly demonstrate the high heterogeneity and complexity of BM-MSC populations. Such a feature should be taken into strong consideration in an analysis of the possible side effects of TKIs. In addition, it is to be underlined again that MSCs are localized in several tissues and data on their possible phenotypic heterogeneity are completely lacking. These findings are certainly important in evaluating the effects of novel selective TK-targeted compounds to be introduced in cancer therapy.

### MSC differentiation into osteoblasts or adipocytes

MSCs, together with HSCs, are generally described as the cells able to initiate BM reconstitution after mechanical injury [83, 84]. Inside the BM microenvironment, MSCs, mainly localized to line the endosteum and in perivascular region, participate to the hematopoietic niche, being essential for HSCs commitment, mobilization, and exit from the marrow compartment and give origin to all BM stromal elements, such as osteoblasts, adipocytes and chondrocytes. In turn, HSCs and their progeny, together with several different cell phenotypes such as endothelial cells, fibroblasts, myocytes, vascular smooth muscle cells, and immune cells, furnish growth factors to BM-MSCs, influencing their stem cell self-renewal or their differentiation abilities.

Within BM, the differentiation of MSCs into osteoblasts is crucial to form bone during development and contribute to bone plasticity and metabolism throughout the life. As described above, osteogenesis embraces two processes: a) intramembranous ossification, due to direct differentiation of MSCs of neuroectodermal origin into osteoblasts, interesting craniofacial bones and part of the clavicle; b) endochondral ossification of axial and appendicular bones, in which osteoblasts, derived from MSCs of mesodermal origin lining the inner face of the pericondrium deposit bone matrix on the partially degraded cartilage and progressively replace hypertrofic chondrocytes, finally removed by apoptosis [85, 86]. Alternatively, osteoblasts might evolve directly from hypertrofic chondrocytes, which do not undergo apoptosis, as it has been recently reported [87].

In both models, MSCs give origin directly, or indirectly, to osteoblasts. Recently, very thorough studies show some differences in the properties of BM-MSCs during development and/or in the post-natal life, in terms of embryonic origin, proliferation/differentiation ability and in terms of cell surface markers used for their identification/classification [72]. In particular, in BM during embrional life Nes^−^ MSCs are in clear majority and actively differentiate into bone-synthesizing osteoblasts substituting cartilage, while the little percentage of Nes^+^ MSCs (of neuroectodermal origin) are almost in quiescence; this ratio is strongly inverted after birth, where Nes^+^ MSCs become important constituents of the haematopoietic niche, being also able to differentiate towards the main stromal cell types and therefore contributing to osteogenesis and to BM adipogenesis.

Osteogenesis begins with the MSCs commitment to osteoprogenitor cells, endowed with high proliferating capacity, which then differentiate into pre-osteoblasts, still able to proliferate. During this active proliferation phase, cells also start to express genes encoding for type I collagen and for other proteins of the extracellular matrix (ECM), such as fibronectin. Also, an increase in the alkaline phosphatase (AP) activity and osteocalcin (OC) synthesis is observed. Pre-osteoblasts then differentiate into osteoblasts; their morphology changes from a fibroblast-like to a roughly cuboidal shape. The cells acquire an abundant rough endoplasmic reticulum, a large Golgi apparatus and plasmatic membrane regions specialized for vescicles trafficking. In fact, these cells actively secrete, in addition to the most abundant type I collagen, several other non-collagen proteins, like OC, osteopontin (OPN), fibronectin and bone sialoproteins. In this phase, high levels of AP are also produced. The ECM then is saturated with crystals of hydroxyapatite, so the matrix results calcified [88].

MSCs can also give rise to adipocytes. Adipogenesis is an extremely ordered process initiating during the development and persisting throughout the life. It can be organized into two main steps: the determination phase, in which MSCs lose their ability to differentiate into other “(mesenchymal) lineages” and the terminal differentiation phase, when the committed preadipocytes mature into spherical adipocytes, capable of synthesizing and storing lipids, releasing adipocyte specific proteins and containing the machinery necessary for insulin sensitivity [89].

AT can be seen as a multi-depot organ distributed in the body endowed with a high physiological plasticity and the overall function of storing energy in form of anhydrous triglycerides droplets [90]. Homeostatic balance between triglycerides storage and mobilization appears important to limit the lipid AT spillover and accumulation in peripheral tissues, which could determine impairment of insulin activity and of pancreatic insulin production (lipotoxicity). In addition, AT is a factual endocrine organ that produces and releases many mediators functioning by autocrine and paracrine behavior, with local and distant effects. AT is organized in White AT (WAT) and Brown AT (BAT). Adipocytes from WAT and BAT diverge by size, abundance of lipid droplets, and number of mitochondria. WAT adipocytes present a single large lipid droplet and scarce number of mitochondria, while BAT adipocytes show several small droplets and increased number of mitochondria. Functionally, WAT stores energy, while BAT dissipates energy for thermogenesis using mitochondrial uncoupling protein-1 (UCP1). BAT was believed to be absent in adult humans; however, recently, it has been clearly demonstrated that functional BAT is also present in specific body sites in adults [91–94]. Furthermore, for long time WAT and BAT adipocytes have been thought as sharing a common developmental origin, until it was recently demonstrated that brown adipocytes have a common precursor with muscle cells, while only WAT derives from MSCs. Very recently, it has also been shown that, although they have different histological origins, WAT can be converted in BAT. Indeed, this feature received great attention, as browning of white energy-storing adipocytes is thought as a good strategy to fight obesity and several related pathologies, such as insulin sensitivity, diabetes and metabolic syndrome [95].

BM adipogenesis is a physiological process. Marrow fat has several functions, including maintenance of the bone microenvironment and energy [96]. It may strongly influence bone remodeling by means of secretion of fatty acids and adipokines, exerting a paracrine actions on stem cells, precursors as well as fully differentiated cell phenotypes, such as osteoblasts and osteoclasts [97]. Also, BM adipocytes may influence HSC differentiation, body energy homeostasis, cancer homing and metastatization [96, 97]. Interesting studies point to the cytokines and hormones produced by BM adipocytes (particularly adiponectin) as important factors promoting insulin sensitivity, fat oxidation, and exerting important antiatherogenic effects [98].

An emerging field of research reached the conclusion that marrow fat is constituted by neither white nor brown adipocytes [96, 97]. Recent studies also exclude the intermediate phenotype of beige adipocytes, initially called into question for the BM fat. Most probably, BM fat may be a non-homogeneous population, probably unique in the pattern of surface markers, with intermediate characteristics between white and beige adipocytes. Interestingly, very recently it has been observed in BM transplanted patients, that a significant proportion (possibly 10%) of marrow adipocytes can move towards peripheral fat depots and, there, act as lipid-storing adipocytes [99].

Numerous pieces of evidence suggest that, during aging, a drift in MSCs differentiation occurs to sustain adipocyte lineage over the osteoblast lineage. This clearly contributes to unbalance the bone formation/reabsorption equilibrium and ultimately to cause the age-related bone loss or osteoporosis. In addition, the majority of pathological conditions (or therapeutic regimens) associated with bone loss, like increased cortisol production, multiple myeloma, osteoarthritis, anorexia nervosa, HIV-associated lipodystrophy and glucocorticoid treatment, might cause enhanced marrow adiposity. In accord to these *in vivo* observations, *in vitro* experiments employing BM-derived MSCs demonstrated that factors inducing adipogenesis might inhibit osteoblast formation and, *vice versa*, factors that promote osteoblastogenesis inhibit adipocyte maturation [89].

Since the global prevalence of osteoporosis and obesity increased strongly in recent years, the inverse relationship between adipocytes and osteoblasts has attracted great attention, also because both cell types are fundamental components of the BM microenvironment. Therefore, intense studies, both *in vivo* and *in vitro*, have been carried out to elucidate the specific pathways involved in each specific MSC lineage. However, as it frequently occurs in science, a definite MSCs differentiation hierarchy is still hypothetical.

*In vitro*, MSCs can be propagated in medium added with 10% serum and glutamine (a so-called standard medium). Upon exposure to different stimuli, these cells are able to differentiate towards all different stromal phenotypes. Specifically, when confluent layer of MSCs are cultured in presence of standard medium enriched with 100 nM dexamethasone, 50 μM ascorbic acid and 10 mM β-glycerophosphate (osteogenic medium, OM), they differentiate into osteoblasts, with a almost cuboidal shape [89]. This cell phenotype (osteoblasts) secrete extracellular matrix (ECM), mostly composed of type I collagen, that can bind calcium salts, so the matrix turns out to be calcified. Alternatively, when these cells are exposed to medium containing 1 μ M dexamethasone, 6 μ M insulin and 0.45 mM 3-isobutyl-1-methylxanthine, they give rise to adipocytes, containing lipid-storing vesicles, evidenced by specific dyes [89].

Commitment of BM-MSCs to the osteoblast or adipocyte fate occurs through a highly regulated mechanism: factors and pathways that promote one cell fate generally suppress the alternative lineage. This articulated process of differentiation is driven by a multitude of stimuli and inhibitors, such as cytokines, growth factors, extracellular matrix molecules, mechanical forces, able to activate different signal transduction pathways, finally ending in the timely orchestrated activation of specific transcription factors. These proteins function as molecular switches able to drive the differentiation of uncommitted precursors towards a specific lineage, controlling the expression of patterns of genes (gene signature) that allow the cells to acquire the specific stromal phenotype [89].

The transcription factors Runx2 and Osx are the main determinants of MSC osteoblastogenesis. In particular, the enhancement of Runx2 in MSCs induces their differentiation into immature osteoblasts, while prevents their commitment to adipocytes [100]. Runx2, however, seems required for the up-regulation of genes encoding the main bone matrix proteins in immature osteoblasts, while it is not essential for the activation of these genes in mature osteoblasts [101]. Conversely, Osx and β-catenin (a Wnt-activated transcription factor) are involved in the final steps of osteoblasts maturation [100, 102], a time period in which Runx2 levels decrease [103].

Adipogenesis requires a completely different scenery of transcription factors. They include, in particular, PPARγ, members of C/EBP family (C/EBPα, C/EBPβ and C/EBPδ) and CREB [104–106]. C/EBPβ and C/EBPδ are rapidly increased after the activation of adipogenic commitment. On the other hand, C/EBPβ requires an obligatory phosphorylation on threonine 179 or serine 184 before to bind to DNA. Following the interaction of C/EBPβ to its specific *consensus* sequences, PPARγ and C/EBPα are up-regulated and maintained at high levels along the whole process of adipogenesis.

### MSCs and hematopoietic niche

As reported above, one of the major roles of MSCs is their participation in the control of hematopoiesis. Hematopoiesis is a very well orchestrated process by which mature blood cells are released into the circulation. Every day, more than 500 × 10^9^ mature blood cells are produced, playing pivotal roles in oxygen transport, immune response and tissue homeostasis. HSCs are the cellular phenotype at the top of the hematopoiesis hierarchy, since they are endowed with key properties such as long-term self-renewal capacity, multipotency, i.e ability to differentiate into all blood cell phenotypes, and, finally, maintenance in quiescence [107].

During development, HSCs derive from the aorta-gonads-mesonephros, particularly from the hemogenic endothelium of the dorsal aorta [108–110]. Studies in mice pointed also to yolk sac as HSCs extra-embryonic origin at early phase of gestation (E10.0) or to the placenta as an important source of HSCs during embryogenesis. Successively, HSCs are released into circulation, moving to an intermediate hemopoietic tissue, such as fetal liver in mammals, where they undergo to rapid expansion and, finally, migrate to the BM, which is the hemopoietic organ in post-natal and in adult life [111]. In BM, HSCs localize in trabecular bones, and rarely in long bones diaphyses [112].

In BM, HSCs and the hemopoietic progenitors reside in specific local microenvironments, termed as hematopoietic niches, which provide signals and regulatory factors essential for HSCs quiescence, localization, self-renewal, proliferation and differentiation. The concept of “hematopoietic stem cells niche”, first proposed by Schofield in 1978, as “an entity in which the stem cell's maturation is prevented and the properties of *stemness* are preserved” [113] has been widely spread, but also intensely debated.

Initial investigations suggested that HSCs were localized in close contact with cells lining the endosteum, i.e. at the interface between mineralized bone and BM inside it [114]. These lining cells were mainly represented by osteoblasts and spindle-shaped N-Cadherin^+^CD45^+^ osteoblasts (SNO), a subpopulation of immature osteoblasts expressing N-Cadherin [115]. N-Cadherins are transmembrane proteins acting as calcium-dependent adhesion molecules to form “adherens junctions”. The endosteum localization of HSCs, together with numerous observations based on *in vitro* (coculture of HSCs and osteoblasts) and *in vivo* studies, pointed to osteoblasts and, among them, to SNO cells [115–117], as major constituents of the hematopoietic niche (endosteum niche). Among the factors produced by osteoblastic cells, OPN is an extracellular protein reported as able to maintain HSCs at level of their niche and to regulate negatively hemopoietic precursors number [118]. Furthermore, osteoblasts produce CXCL12, which is essential for HSCs maintenance in BM and quiescence [119], and angiopoietin-1 and thrombopoietin, which, through binding to their receptors, Tie2 and MPL respectively, promote the quiescence of HSCs [107, 120, 121]. N-Cadherin^+^-osteoblasts were thought to interact directly by omophilic adhesion with N-Cadherin^+^-HSCs, but this matter has been deeply dibated, since it was not clear if HSCs express N-cadherins [122, 123]. Osteoblasts however seem also associated to HSC proliferation, particularly through activation of the Notch pathway signalling [124].

The composition of hemopoietic niche was subsequently questioned by studies on mice in which the osteoblast number was reduced, but hematopoiesis appeared not affected [125, 126] Accordingly, hematopoiesis still remained grossly normal when SCF, a growth factor essential for HSC maintenance, was conditionally deleted in the osteoblast lineage [127]. Finally, additional experimental evidence, including conditional deletion of *CDH2* (encoding for N-cadherin) in osteoblast lineage cells, argued for a dispensable role of N-cadherin in HSC maintenance and function [126]. These data, however, did not directly exclude the involvement of SNO cells in hematopoiesis.

A more accurate characterization (in terms of antigen markers) of HSCs, along with highly sophisticated imaging analyses, have allowed to detect higher density of HSCs in a position proximal to sinusoid vessels, which are highly fenestrated specialized venules, where they interact directly or indirectly with several cell populations. This allowed the emerging of the concept of the “perivascular hematopoietic niche” [128]. The perivascular localization of HSCs focused the attention on endothelial and stromal cells as fundamental constituents of this HSC niche, with the major involvement of the cells embracing the sinusoids, but with a niche function for cells surrounding the arterioles, too. Considering that the endosteum is highly vascularized, particularly by a network of arterioles, the two apparently distinct concepts of endosteal and perivascular niche might converge, in that HSCs could be mainly localized around vasculature and particularly in the endosteum area [107]. Numerous studies demonstrate the involvement of bone marrow sinusoidal endothelial cells in supporting HSCs maintenance (by means of SCF, angiopoietin and other cytokine expression) and proliferation. Indeed, knock-out of the endothelial-specific adhesion molecule E-selectin, caused an increase of HSCs number in quiescence and inhibition of proliferation [129]. In addition to endothelial cells, the perivascular region contains a heterogeneous population of mesenchymal stem cells and stromal cells, and also embraces adipocytes, neurocytes and glial cells. Given their localization, these cell populations have all been studied for their interaction with HSCs and for their putative role as hematopoietic niche cells [83].

It has been definitely established that CXCL12 is a cytokine that plays a fundamental role in HSC homeostasis and is absolutely required for HSC niche activity [119]. As a matter of facts, the signaling activated by the interaction of CXCL12 with its receptor CXCR4 (expressed on cells of hematopoietic origin) is essential for homing and maintenance in BM of HSCs and precursors of B cells, plasmacytoid dendritic cells (pDCs), and NK cells [120, 130]. Consequently, the characterization of CXCL12-producing cells represented a strategy for the identification of BM cells involved in the hematopoietic niche structure.

Genetic Cre tracing studies (see also the paragraph on BM-MSC populations and lineage tracing studies) revealed that CXCL12 is expressed in CAR cells, Nes^+^ stromal cells, and LepR^+^ stromal cells [131, 132]. A partial overlapping among these cell populations has also been clearly reported, although the occurrence of CXCL12^+^Nes^−^ cells has been demonstrated [131].

CAR cells (see the paragraph “BM-MSC populations and lineage tracing studies”) are mesenchymal perisinusoidal reticular cells with long cellular processes [120, 133]. These cells can directly interact with HSCs and their offspring or with other cells in proximity. CAR cells are endowed with the ability to fully differentiate *in vitro* in osteoblasts or adipocytes [83], and also to produce the highest levels of CXCL12 and SCF factors, fundamental for HSCs function maintenance. The role of these stromal cells in hemopoiesis has been investigated in detail by means of their conditional ablation, obtained by short-term diphtheria toxin treatment of transgenic mice expressing the diphtheria toxin receptor (DTR) under control of CXCL12 promoter (CXCL12-DTR mice). The treatment caused a reduction in HSCs number and in HSCs repopulating activity, but an increase in HSC quiescence [81]. This methodology, however, does not exclude an ablation of other distinct populations expressing CXCL12, such as osteoblasts or endothelial cells, although no macroscopic toxicity was observed for these cell phenotypes in CXCL12-Dtr mice.

Very recently, the use of Ocn-Cre, that specifically target osteoblasts, has been reported to target also 72 ± 4.0% of CAR cells and a subset of NG2^+^ arteriolar pericytes [134]. Similarly, Dmp1 (Dentin matrix acidic phosphoprotein 1)-Cre has been used to target specifically osteocytes, since Dmp1 is expressed specifically in this phenotype. However, Dmp1-Cre also efficiently targets approximately 40% of CAR cells [134]. This suggests a remarkable heterogeneity of CAR cells and the existence of a complex interplay between CAR cells, MSCs populations and their progenies.

Nes^+^ cells have been also implicated in HSC homeostasis, since they express CXCL12, SCF, and angiopoietin, all factors sustaining HSCs maintenance. These cells have CFU-F activity, form mesenspheres and are able to differentiate towards osteoblasts, adipocytes and chondrocytes. [131]. Also, it has been observed that transplanted HSCs home principally in proximity of Nes^+^ cells [131]. As above mentioned, more recently Nes expression has been found in two populations, one in periarterioles position (rare, but with higher Nes levels) and the other around the perisinusoids [76]. Both populations have CFU-F ability. However, the analysis of transcriptome revealed that periarterioles Nes^+^ cells show a higher expression of genes associated with the HSC niche cell function and reduced expression levels of genes associated to cell cycle progression and proliferation. Indeed, they have also been implicated in harboring quiescent HSCs and are themselves in an unreplicative status [76].

Another population expressing high levels of CXCL12 and SCF and showing perisinusoidal localization is represented by LepR^+^ cells, identified by lineage tracing studies using Cre recombinase under control of LepR promoter [127]. Particularly, deletion of SCF (Kit1) in LepR^+^ cells determines HSCs reduction, suggesting the importance of these cells in HSCs homeostasis. Also, several pieces of evidence indicate that the most abundant LepR^+^ cells, together with CAR cells, with which they have some degree of overlapping, may represent the major source of bone and fat cells in adult BM, as well as a key source of hematopoietic niche factors [135, 136]. In fact, CAR cells isolated by cell sorting were reported to express high levels of LepR [131] and also CAR cells are able to differentiate *in vitro* into adipocytes or osteoblasts, similarly to LepR^+^ cells.

An additional important feature of the niche microenviroment strongly modulating its activity is the noteworthy low pO_2_ pressure that ranges about 8-9% [137]. This relative hypoxia triggers molecular mechanisms able to stabilize and activate pivotal transcription factors, mainly the members of HIF-α functionality family [HIF-1α and HIF-2α) [138]. HIF-α proteins might modulates, in a phenotype-dependent context, the processes of proliferation, differentiation and apoptosis [138]. Intriguingly, alterations of HIF-α levels have been correlated to human hereditary pathologies showing polycythemias [139–141]. In addition, it has been recently demonstrated that low pO_2_ stimulates CML cells clonogenicity and their capability to repopulate immunodeficient mice. These effects occur independent of IM treatment and might involve, at least in part, a down-regulation of HIF-1α activity. Thus, also the expression of BCRAbl1 (and the resistance to IM treatment) appears controlled by HIF-1α levels and, thus, by pO_2_ microenvironmental level [142].

In conclusion, although the role of MSCs in hemopoietic niche is clear, the complexity of populations does not allow the construction of a simple model. What appears clear is the occurrence of various phenotypes, each expressing, at different degree, the most intriguing markers. Importantly, while most of the investigations have been performed in mice, the data available in humans are scarce.

In human BM, CD146^+^ cells represent a MSC-rich population [143]. Recently, a BM stromal cell population expressing PDGFRα^+^ and CD51^+^ has been identified, in mice and humans, as enriched for MSCs and able to sustain *in vitro* HSC expansion [79]. Human CD146^+^ skeletal stem cells localize in BM near to sinusoids and are able to express high levels of SCF and CXCL12. These studies provided some evidence that in humans CD146^+^PDGFRα^+^CD51^+^ cells are one key MSC component of a perivascular niche for HSCs.

A fascinating and intriguing aspect of the complex interplay between MSC and the hematopoietic niche microenvironment, required for a correct HSCs activity, is the emerging hypothesis that leukemic cells might modulate BM-MSC populations equilibrium to gain growth advantage for cancer cells. It has been reported that BM-MSCs from acute myeloid leukemia (AML) patients present diminished osteogenic and enhanced adipogenic differentiation [144]. In particular, it has been proposed that this unbalanced commitment might give a disadvantage to physiological hematopoiesis and a relative advantage to malignant leukemogenesis [144]. The study has however not been confirmed [145]. As a matter of fact, in this distinct report, the authors presented evidence suggesting that MSCs from AML patients (AML-MSCs) are characterized by a more pronounced osteogenic phenotype, as showed by the increased expression of osteogenic specific markers, as Runx-2, osteopontin, tissue nonspecific alkaline phosphatase (TNAP) and osterix [145]. The increased osteogenic differentiation of AML-MSCs was also confirmed in a mice model of AML. The mechanism by which AML may force osteogenic differentiation involves the initial production by leukemic cells of BMP (bone morphogenetic protein) that acts on MSCs. Then, MSCs increase the production of connective tissue growth factor (CTGF) that facilitates the proliferation of AML cells [145]. Although the precise effect of leukemia cells on MSCs is not well established, the general conclusion is that there is a reciprocal influence, where AML affects MSCs and, in turn, MSCs enhance AML cells proliferation.

It has also been described that BM microenvironment of patients affected by Acute Lymphoid Leukemia (ALL) is capable of inhibiting the osteoblast-dependent sustenance to HSC physiological function [146]. Also in this case, the mechanism was associated to a reduced osteoblast differentiation, although an increase of adipogenesis was not reported. In this setting (i.e. ALL), the molecular mechanisms involve an aberrant Notch activation that causes a reduction of CXCL12 expression in osteoblasts and an altered osteoblastic differentiation.

Therefore, although the idea that changes in MSC populations may be major players of the altered hematopoiesis in leukemias is extremely appealing, the suggestion still needs, in our opinion, further confirmation. In addition, being leukemias treated frequently with TKI, it should be also considered the possibility that the observed MSCs alterations are due (at last in part) to TKIs treatment and not to a direct influence of leukemic cells.

Finally, it is to be underlined that not only an effect of leukemic cells on MSCs has been described, but also the possibility that MSCs dysfunction might induce leukemogenesis [147]. Indeed, it has been reported that deletion of Dicer1 in mouse osteoprogenitor cells can disrupt the integrity of haematopoiesis and induces myelodisplasia and AML. The hypothesized mechanism involves the reduced expression (due to Dicer1 ablation) of SBDS, the gene mutated in the Shwachman-Bodian-Diamond syndrome, in MSCs and the subsequent alterations of HSCs homeostasis [147]. Therefore, a primary stromal genetic alteration might give origin to a secondary neoplastic disease, sustaining the new and intriguing concept of “niche-induced leukemogenesis”.

### MSCs as immunomodulatory cells

Numerous *in vitro* investigations, along with studies carried out in pre-clinical models, have demonstrated that MSCs show important immunomodulatory activities useful for the treatment of immunological-associated diseases. These findings have found a clear confirmation in a significant number of clinical trials [148, 149]. Although the aim of this review is not to report a detailed list of clinical experiments demonstrating the ability of MSCs to reduce the immunological response, it seems useful to briefly summarize some critical reports.

In 2000, Koc *et al*. prepared MSCs from BM aspirates of breast cancer patients. Then, they re-injected cell preparations plus autologous PBPCs (i.e. Peripheral-blood progenitor-cells) in patients given high-dose chemotherapy and granulocyte colony-stimulating factor. The authors demonstrated, for the first time, that autologous MSCs infusion at the time of PBPC transplantation was feasible and safe [150]. Moreover, the study reported a rapid hematopoietic recovery, indicating that MSC infusion may have a positive impact on hematopoiesis [152]. In 2002, in a short report on the “British Journal of Haematology”, Lee *et al*. described the case of a patient affected by AML and transplanted with mobilized hematopoietic stem cells in combination with MSCs prepared from an HLA-haploidentical donor [151]. Interestingly, no relapse was observed up to 31 months after transplantation. Subsequently, an increasing number of studies demonstrated that MSCs were able to statistically ameliorate the engraftment of hematopoietic staminal cell transplantation, and very importantly, to reduce the graft *versus* host disease (GVHD) [152, 153].

MSCs are clearly able of communicating with the microenvironment during the immune response. Such a connection consists in a strict interaction and cross-talk either with cells involved in the innate or adaptive immunity [149, 154]. As a matter of a fact, MSCs might express on their surface a number of proteins of the integrin and adhesion families that induce interactions with cells of the immunological system. Moreover, MSCs are regulated by the inflammatory milieu, indicating that the immunodulatory capabilities of MSCs are not constitutive, but are induced by the cytokines produced by other activated immune cells. Since the response to cytokines critically influences the immunological behavior of MSCs, it is evident that the immunomodulatory properties of these cells might be considered as “plastic” [155].

A pivotal aspect should be also underlined, i.e. that the positive responses were independent of the source of human MSCs. This suggests the possibility of banking of large amounts of MSCs for their clinical use.

It is important to state that MSCs do not constitutively express either MHC class II proteins or costimulatory molecules (C40, CD80 or CD46). Conversely, during the inflammatory response, MSCs up-regulate the expression of MHC class II, even in the absence of T cell activation [156]. Generally speaking, MSCs are able to activate a number of tolerogenic mechanisms that involve the major mechanisms of immunity, namely cell-to-cell contact, modulation of immunological cells, release of cytokines and activation of anergy. The mechanisms that have been considered for explaining the immunomodulatory effects of MSCs are quite heterogeneous, and practically, involve all the major steps of immune response. In brief, these might be summarized as reported in the following points.

1. MSCs might regulate the function of dendritic cells (DC). In general, MSCs modify the phenotype (namely, the release of cytokines and maturation) of DCs and negatively affect their ability of antigen presentation [157]. In turn, DCs cannot efficiently prime T cells for a strong immune response.

2. MSCs alter the processes of lymphocyte activation, proliferation and differentiation. In general, MSCs mainly induce the activation as well as expansion of subsets of T-cells that harbor peculiar regulatory phenotypes. Moreover, the phenotype of T-reg (T regulatory) and their functions are maintained after the recruitment of MSCs [158, 159]. Additional experiments showed that MSCs stimulate the origin of T-regs starting form CD4+/CD8+ lymphocytes. Such T-regs have the capability of inhibiting powerfully the activation of lymphocytes [160, 161]. Contemporaneously, MSCs induced T-regs suppressing activated T cells [162]. In this context, it is also to underscore the occurrence of cross-regulation between MSCs and T-regs. As a matter of fact, the contemporaneous transplantation of T-regs with MSCs results in a significant increase (evaluated as rate of survival, proliferation and angiogenesis) of MSCs. This cross regulation (MSCs increase T-regs and *vice versa*) has positive effect for cell-based treatments [165]. Finally, although the positive effects of MSCs on T-regs appear quite clearly established, the precise molecular mechanisms that are at the bases of this activity have not been definitely delineated.

3. A growing number of reports have clearly pointed to the humoral immune response and the B cell populations as key mediators in the process of chronic rejection of allografts. Indeed, MSCs have been described as capable to affect the activity of B-lymphocytes and, thus, to modify the humoral immune response. To date, the classification of B cells into different subsets has been linked to distinct profiles of cytokine secretion. A new intriguing B cell population, defined as B regulatory subset or B-reg has been identified. This B cell subset appears particularly important for the induction and/or maintenance of tolerance [164]. Significantly, the absence or reduction of B-regs appear associated to the development of a number of autoimmune diseases, at least in a mouse experimental model [165]. The percentage of B-regs is extremely scarce, but they can be expanded *in vitro* under specific conditions. MSCs appear able to increase the amount and activity of B-regs and, in exerting this activity, MSCs ameliorated the symptoms of systemic lupus erythematosus in a murine model of the disease. The positive effect on B-regs might also be correlated to the immunomodulatory activity of MSCs on chronic GVHD [166].

4. Finally, MSCs affects the functions of NKs (natural killer cells). NKs are cytotoxic effector cells and are a major component of the innate response. These cells play a critical role either in innate immunity or in adaptive response. As a matter of fact, NKs participate to the reaction against the allograft, being able to recognize *self* from *non-self* and to lyse the potential targets [167]. MSCs inhibit activated NKs and hamper their proliferation as well as their capability of releasing specific cytokines. In addition, MSCs affect the NK phenotype by reducing the expression of markers characterizing their activation (CD132, NKG2D, NKp30 and NKp44) [168, 169]. In brief, a definite immunomodulatory activity of MSCs is characterized by a clear reduction of NK activity. An intriguing MSCs function is the induction of a specific NK subset (CD73^+^ NK) that is able to create a tolerogenic microenvironment with a low level of inflammation [170].

## MSC AND TK INHIBITORS

As described in the previous paragraphs, the role of MSCs is of great relevance in a variety of physiological processes. These cells show promising perspectives as important immunomodulatory agents in several clinical settings, including BM transplantation and GVHD. Analogously, TKIs are undoubtedly pivotal therapeutics for treatment of human cancers and non-malignant diseases. Nevertheless, the effects of TKIs on MSC phenotypes have been investigated in a relatively low number of instances and only for very few inhibitors. Intriguingly, the data obtained are frequently conflicting, so that a definite picture is not available, yet.

Definitely, the large majority of studies have been performed employing BCR-Abl1 inhibitors. This is probably related to the circumstance that IM is the TKI prototype and one of the most successful molecularly targeted anticancer compounds. In the following paragraph, the effect of BCR-Abl1 inhibitors will be analyzed in more detail. Then, we will describe the data available on other TKIs, either approved or not by FDA for therapy.

### BCR-Abl1 inhibitors and MSCs

The effect of all major BCR-Abl1 inhibitors, i.e. IM, nilotinib, dasatinib and bosutinib, on MSCs phenotype has been investigated in several instances. The addition of IM to *in vitro* cultures of human MSCs results, in all the reported studies, in an inhibition of growth. To the best of our knowledge, the first report of the IM effects on human MSCs was published by Fierro and colleagues in 2007 [171]. The authors described that IM not only inhibited MSC proliferation, but also favored adipogenesis, while contemporaneously suppressing osteogenesis [152]. The proposed mechanism of action includes the inhibition of PDGFRβ and the reduction of AKT and Erk1/2 activity [171]. In 2007, the effect of IM on rat osteoblasts was also described, but in this case the TKI was reported to induce increased mineral calcification by promoting osteoblast differentiation and osteoclast inhibition [172]. The molecular effect proposed also involves PDGFRβ inhibition [153]. In 2008, Fitter and colleagues confirmed that IM inhibits human MSC proliferation, but, surprisingly, reported that the molecule stimulated mineralized matrix deposition and increased the osteogenic gene expression [48]. The mechanism was identical to that previously reported by Fierro *et al*., namely the inhibition of PDGF receptor function and AKT activity. In particular, the authors stimulated the cells by PDGF and observed that IM inhibited in a dose-dependent fashion AKT and CRK-L activity [48]. In addition, to confirm their results, Fitter *et al*. reported that pharmacologic inhibition of PI3-kinase/Akt promoted mineral formation [48].

In a subsequent study, Tibullo and colleagues (2009) [173] cultured for 21 days human BM-MSCs in presence of OM plus or minus 1 μM IM. They observed that IM increased the formation of an extensive network of dense multilayered nodules characteristic of enhanced extracellular mineralization [154]. In addition, IM elevated the expression of osteogenic markers, such as Runx2, Ocn, and BMP-2. In accord, Ocn level and Opg/RANKL (osteoprotegerin/receptor activator of nuclear factor-kB ligand) ratio increased in the supernatant of MSCs treated with IM [173]. On the other hand, in 2010, Fitter and coll. described that, in CML patients, 6 months of IM treatment resulted in the increase of BM adipocytes and in a 3-fold up-regulation of adiponectin serum levels [174].

In 2012, two additional studies investigated the activity of IM on MSCs [176, 177]. Jonsson *et al*. confirmed the antiproliferative activity of the molecule and the TKI-promoted MSC osteoblastic commitment [175]. However, they found a drug biphasic effect in that, while IM stimulates an early osteoblastic differentiation (as estimated by alkaline phosphatase activity), then it reduces the ECM mineralization (evaluated by Alizarin Red staining). In addition, the authors observed the Runx2 transcript increase (at early time points) and the osterix mRNA down-regulation (late in differentiation). They concluded that the effect of the BCR-Abl1 inhibitor also depends on the maturation stage of the cells [175]. In the same year, Fitter and colleagues reported that IM promotes adipogenesis of MSCs by PDGF inhibition and reduction of PI3 kinase [176]. In addition, the IM-stimulated adipocytes secreted adiponectin, that might account for the improved glucose and lipid metabolism in CML patients concurrently affected by type 2 diabetes treated with IM [177–180]. All together, these studies allow the drawing of one major conclusion, i.e. that IM inhibits MSCs growth. Conversely, whether the drug induces osteoblastic or adipogenic differentiation is not clear and probably might depend on the experimental conditions employed in every specific experiment. Most recently [181], it has been reported that a mouse MSC cell line (OP9 cells) treated with different TKIs (including IM, dasatinib and sunitinib) acquired a novel functional status, changing the expression of several genes, some of those encoding for adhesion molecules, growth factors and chemo-attractants. The investigation was not primarily aimed to identify the phenotypic effects of IM (and additional TKIs) on MSCs. However, it, indirectly, confirms that these cells are targets of TKIs and that the response to the drug(s) is remarkably complex [181]. The study, on the other hand, characterizes a novel sequence of events activated by TKIs. Particularly, TKI-treated MSCs express a variety of adhesion molecules, prosurvival growth factors and chemo-attractants that allow leukemic cells to form clusters near MSCs. Such clusters allow the leukemic cells to survive the pharmacological treatment. The mechanism of survival is linked to the activation of IL-7R/Janus kinase signaling, that can substitute the BCR-Abl1 signaling pathway inhibited by the drug [181]. The most fascinating results were, however, mainly obtained by *in vitro* experiments and thus needs further confirmation [181].

Second-line BCR-Abl1 inhibitors have also been investigated for their activity on MSCs. The effects of nilotinib were analyzed for the first time in 2011 [50]. It was found that the TKI potently inhibited primary rat osteoblastic proliferation through PDGFR inhibition. The molecule also reduced osteoblastic differentiation at low concentration (0.1-0.5 μM) while, conversely, had no effect on the phenotype at 1 μM. Nilotinib also increased expression and secretion of osteoprotegerin and decreased expression of RANKL. Few months later, in a letter to Bone, these data were confirmed employing human MSCs [182]. The authors showed that nilotinib inhibited MSC osteoblastic differentiation through reduction of alkaline phosphatase activity and of matrix mineralization (seen by Alizarin-red S stain and von Kossa's stain). To further confirm the effects of nilotinib, the authors reported that nilotinib reduced Runx2 and Osx transcripts as well as bone sialoprotein (BSP) and Opn [182]. They also suggested that the activities of the TKI were due to c-Abl or DDR1 kinases inhibition. In contrast with these data, the following year, Tibullo and coll. [183] reported that the addition of dasatinib, and to a greater extend nilotinib, to the OM induced expression of osteogenic mRNA markers (i.e. RUNX2, Ocn and BMP2) and increased the Opg/RANKL ratio. The 2015 study, described above, confirmed the effects of nilotinib on several features of MSCs [181].

The first studies on the effect of dasatinib on MSCs were published in 2010 [184, 185]. Jonsson and coll., in an investigation published on Leukemia, stated that the molecule inhibits the growth of MSCs and their osteoblastic differentiation [184]. The antiproliferative activity was correlated to the inhibition of c-Abl, PDGFR, c-src and other kinases [184]. On the other hand, a different group reported that dasatinib stimulated osteogenesis increasing alkaline phosphatase activity and upregulating the expression of bone sialoprotein and Opn [185]. Moreover, the molecule induces calcification of the matrix and increases the Opg/RANKL ratio [185]. The subsequent year, Borriello and colleagues reported that dasatinib induces adipocytes differentiation of human BM-MSCs [186]. The authors also demonstrated that the molecule up-regulates a number of genes correlated to adipogenesis, including PPARγ, CEBPα, LPL and SREBP1 [186]. Dasatinib, added to OM, also inhibited the activity of alkaline phosphatase and remarkably reduced matrix mineralization [186]. A following study, performed on human BM-MSCs and two cell lines (hMSC-TERT and the MG-63 cell lines) demonstrated that dasatinib induced osteoblastogenesis, again confirming the unusual conflict between data in Literature [187]. In few instances, bosutinib was investigated, but no significant effect of the molecule on MSCs differentiation was evidenced [181].

### Gefitinib, erlotinib, sunitinib and vatalanib

The effect of gefitinib (an EGFR inhibitor) on MSCs has been investigated in at least 3 studies. In 2005, Normanno and coll. reported that human BM-MSCs express immunoreactive EGFR (188). The data were then confirmed in two MSCs-like cell lines, namely, HDS-1 and HDS-2 cells. When these cells were treated with gefitinib, a reduction of EGFR activation was evidenced, along with Akt down-regulation. In turn, this inhibition resulted in an evident reduction of released M-CSF (macrophage colony-stimulating factor) and RANKL. This caused a reduced osteoclast stimulation. In a subsequent study, the same research group investigated a cross-talk existing between prostate cancer cell line (PC3 cells) and MSCs and demonstrated again that some physiological functions depending on the EGF-activation of MSCs (i.e. CCL5 secretion) might be inhibited by gefitinib [189]. Contemporaneously, Liu *et al*. (2013) reported a study in which MSCs were induced to differentiate by the addition of BMP9 and EGF (EGF potentiates the activity of BMP9). The authors concluded that gefinitib and erlotinib (or other receptor TKIs, AG-1478 and AG-494) were able to inhibit MSCs differentiation in a dose- and time-dependent manner by interfering with a synergic cross-talk between EGF and BMP9 pathways [190]. The authors also suggested that EGFR expression was increased by BMP9 through Smad signaling pathway [190].

Few other data exist on the effect of erlotinib (Tarceva, a EGFR inhibitor) on MSCs. In particular, in the already cited 2015 paper, the molecule was tested, along with IM, dasatinib and sunitinib (181), and showed to be less active than the other compounds in modulating the interaction of MSCs with leukemic cells [181].

## CONCLUSIONS AND FUTURE DIRECTIONS

Nearly all the human tissues harbor a mesenchymal stromal cell population possessing stem cell-like features, including wide differentiation capacities and self-renewal properties. Although the characterization of their distribution is undoubtedly incomplete, MSCs have been demonstrated, in addition to BM and AT, in several adult tissues, including peripheral blood, lung, liver, skeletal muscle, skin and heart. Analogously, several neonatal-associated tissues are rich of MSCs (umbilical cord, placenta and cord blood). These MSCs are generally located in specific niches and, on the basis of their potentiality, play several roles including, after an appropriate commitment, the regeneration of altered tissues. The other side of the coin is that an altered proliferation/commitment/differentiation of these cells might results in pathological conditions.

In liver, when moderate injury takes place, the regeneration is accomplished by local hepatocytes. Liver MSCs participate to the process, with a clear cooperation between different types of stem cells to preserve cell balance and tissue homeostasis. On the other hand, liver MSCs might give rise to a fibrotic process when this delicate balance is altered [191]. Similarly, skeletal muscle MSCs (also known as satellite cells) participate to muscle regeneration acting as stromal/progenitor cells allowing the repair of injured myofibers. However, under disease conditions, these MSCs might play a “sinister role”, in that they represent a major source for fibrosis, extracellular matrix protein deposition, and fatty tissue in patient affect by dystrophies. Analogous considerations might be reported for MSCs occurring in additional human tissues suggesting a unique molecular “director”, although employing apparently different “cellular actors” [192].

The data available so far demonstrated that inhibitors of tyrosine kinases might affect the metabolism and functions of BM-MSCs. The high heterogeneity of this cell population, largely proved by a number of intriguing and important investigations, suggest that TKIs might act on specific sub-populations, although no data are still available. The effects of such a targeting are not clear in that conflicting results have been reported. However, what appears evident is that these powerful molecules might cause changes in MSCs commitment favoring a specific differentiation. Whether these effects might occur on MSCs sub-populations of tissues different from BM has not been investigated, but is highly probable.

TKIs represent undoubtedly a key class of anticancer drugs whose potentiality has been exploited only partially. Definitely, their efficacy in pathologies different from cancer has been clearly reported. First of all, a positive activity of specific TKIs (namely imatinib, masitinib and atorvastatin) on chronic inflammatory and autoimmune diseases has been reported in several studies [193–200]. In particular, systemic lupus erythematosus, rheumatoid arthritis, psoriasis, inflammatory bowel diseases (primarily Crohn's disease and ulcerative colitis) appear as pathologies that have a benefit from TKIs treatment [201]. Although the majority of results have been obtained in experimental models, a number of initial trials have been already performed confirming the data reported in animals [193–201]. Importantly, also other chronic inflammation-associated conditions, like cardiac hyperthrophy, pulmonary hyperthension, lung fibrosis, atherosclerosis, in-stent renstenosis and glomerulonephritis, have been suggested as responsive to TKI therapy [202–206]. Some TKIs (mostly imatinib and dasatinib) show remarkable promises in the treatment of some metabolic alterations including, in particular type II diabetes. As a matter of facts, the imatinib anti-diabetic activity has been confirmed in several instances [182, 207–209]. Intriguingly, nilotinib seems to have a negative effect on the disease [210]. A third group of human pathologies that appear to positively respond to TKI therapy includes neurological diseases. In particular, ischemic brain stroke, multiple sclerosis, Alzheimer's disease, amyotrophic lateral sclerosis appear to be (at least in part) positively respond to TKI treatment. In these cases, the most effective TKIs appear to be imatinib, masitinib, bosutinib, sorafenib, lestaurtinib [207–215]. In all the above mentioned diseases, the rationale of TKI use is the evidenced involvement of tyrosine kinase targeted in the specific disorder [216]. As an example, a number of studies have involved Syk activity in rheumatoid arthritis, multiple sclerosis, systemic lupus erythematosus and other autoimmune diseases [216].

In conclusion, the ample therapeutcal perspectives of TKIs suggest that their effects on MSCs properties (including MSCs of BM and additional tissues) will represent, a probable source of unexpected side effects in long TKI-treated patients and a key issue of future basic and clinical investigations.
